# Exclusive breastfeeding and women's psychological well-being during the first wave of COVID-19 pandemic in Italy

**DOI:** 10.3389/fpubh.2022.965306

**Published:** 2022-08-23

**Authors:** Claudia Ravaldi, Laura Mosconi, Alyce N. Wilson, Lisa H. Amir, Roberto Bonaiuti, Valdo Ricca, Alfredo Vannacci

**Affiliations:** ^1^Perinatal Research Laboratory, PeaRL, CiaoLapo Foundation for Perinatal Health, Department of Neurosciences, Psychology, Drug Research and Child Health, University of Florence, Florence, Italy; ^2^Maternal, Child and Adolescent Health Program, Burnet Institute, Melbourne, VIC, Australia; ^3^Judith Lumley Centre, School of Nursing and Midwifery, La Trobe University, Bundoora, VIC, Australia; ^4^Breastfeeding Service, Royal Women's Hospital, Parkville, VIC, Australia; ^5^Psychiatry Unit, Department of Health Sciences, University of Florence, Florence, Italy

**Keywords:** COVID-19, breastfeeding, PTSD, anxiety, psychological well-being

## Abstract

**Background:**

At the onset of the COVID-19 pandemic, support for breastfeeding was disrupted in many countries. Italy was severely impacted by the pandemic and is known to have the lowest exclusive breastfeeding rate of all European countries. Considering the inverse association between anxiety and breastfeeding, maternal concerns about the COVID-19 emergency could reduce breastfeeding rates. The aim of the study is to explore the association between infant feeding practices and maternal COVID-19 concerns.

**Methods:**

This paper is a secondary analysis of the cross-sectional study COVID-ASSESS conducted in Italy in 2020. The original survey was administered in two phases: during the first lockdown and during the reopening. The survey included five sections: socio-demographic, medical history, concerns about the COVID-19 pandemic, infant feeding practices and psychometric evaluation. Participants were considered eligible for the *post-hoc* analyses if they were exclusively breastfeeding or they were feeding with infant formula (either alone or with breastfeeding) at the time of the interview.

**Results:**

Between phase 1 and phase 2 there was a decrease in anxiety and concerns about the danger of COVID-19 to general health, except for concerns about their baby's health. Women using formula were more concerned about all the health topics investigated. Moreover, they showed higher levels of stress, state anxiety, somatization and PTSD symptoms.

**Conclusion:**

Breastfeeding during the first pandemic lockdown in Italy seems to have been an independent factor associated with lower anxiety about COVID-19, fewer psychopathological symptoms, and a positive experience of infant feeding.

## Statement of significance

Pregnancy and childbirth care had receded to the background due to COVID-19 pandemic. During this health emergency exclusive breastfeeding rates have declined in Italy, a country which already had one of the lowest rates in Europe. The literature suggests that anxiety in postpartum period negatively influences exclusive breastfeeding, and maternal concerns about the pandemic could have worsened the issue. Moreover, the postpartum period is a delicate stage in women's life due to a greater vulnerability to mental health disorders. There is some evidence that breastfeeding is associated with enhanced maternal physical and mental health compared to formula-feeding. This is the first study to explore the association between maternal COVID-19 concerns and infant feeding practices. Our results show that breastfeeding seems to be an independent protective factor that can promote better mental health status in mothers and a positive experience of infant feeding.

## Introduction

The first cases of COVID-19 were identified in Italy at the end of February 2020. A period of full lockdown followed, and the priorities of the public health system were mainly focused on protecting the most vulnerable and managing patients severely affected by COVID-19. Pregnancy and childbirth were receded to the background, and despite the World Health Organization's recommendations to continue to promote early breastfeeding and skin to skin contact, these were not followed in many settings (1).

Breastfeeding could be a lifesaving intervention for babies, and has been shown to confer short and long-term benefits, e.g., protection against infections, increased intelligence, decreased incidence of overweight and diabetes (2). Moreover, it gives protection to nursing women against breast cancer, ovarian cancer, type 2 diabetes and postpartum depression (2, 3). The WHO European Regions have the lowest global breastfeeding rates (4). In particular, research conducted in 2019 showed that Italy had one of the lowest rates of exclusive breastfeeding of the European countries surveyed (5). Data from the Italian National Health Institute showed that only 23.6% of infants between 4 and 5 months are exclusively breastfed since birth, and this number significantly changes from northern (44.7%) to southern (16.6%) Italy (6). Furthermore, 11.7% of infants had never been breastfed (6). This is a multifaceted issue: women are more likely to exclusively breastfeed at 3 months if they are more educated, resident in the northern or center of Italy, have attended antenatal classes and groups about breastfeeding, have practiced skin to skin contact and have initiated breastfeeding early (7).

In the first weeks of the emergency, there was no evidence about the possibility of vertical transmission of SARS-CoV-2 through breastfeeding or human milk and the literature on vertical transmission of other coronaviruses (MERS or SARS) was very poor (8–10). Several studies showed how this uncertainty affected breastfeeding rates (11). The authors of an Italian study enrolled 204 mothers during the first lockdown and compared them to a group of 306 mothers who took part in a previous study. They collected breastfeeding rates at hospital discharge, 30 and 90 days postpartum and compared them with those of the control cohort. During the lockdown, exclusive breastfeeding at discharge was reported by 69.4% of mothers compared to 97.7% of the control group. Both cohorts showed a decrease at 30 days and 90 days, but the “lockdown group” displayed a dramatic decrease especially at 90 days (31.8 vs. 70.5%) (12).

Postpartum women are particularly vulnerable to mental health disorders, like depression or anxiety, which are one of the major causes of disability during and after pregnancy (13). Results of a Belgian study conducted on pregnant and breastfeeding women during the first months of 2020 showed higher levels of generalized anxiety and major depressive symptoms compared to estimates prior to the pandemic (14). These findings are similar to studies conducted in Canada, Ireland, Norway, Switzerland, the Netherlands, and the UK (15, 16) and suggest that perinatal health should be regarded as a priority issue, especially in the first year after birth.

Although the pandemic likely affected the mental health of mothers of newborns, there is some evidence that breastfeeding is associated with enhanced maternal physical and mental health compared to formula-feeding mothers (17). To better understand the correlation between COVID-19 concerns and infant feeding, we performed a *post-hoc* evaluation of the national survey COVID-ASSESS (18) that evaluated mental health status in two different phases: during the first lockdown for COVID-19 in Italy and during the successive reopening.

## Methods

COVID-ASSESS was a cross-sectional study based on a survey administered in two phases: during the first lockdown for the COVID-19 pandemic in Italy (phase 1) and during the reopening (phase 2). The survey was distributed *via* CiaoLapo, an Italian charity for perinatal loss support, using existing networks and support groups across Italy. Participants self-selected to complete the survey and participation was voluntary. The survey was launched in March 2020, and data were collected until May 2020. Human research ethical approval to conduct the survey was received from Florence University Ethics Committee (Prot. n. 006897). Each participant gave their explicit consent in an online form before enrolment. The survey was uploaded as an online tool using the Surveymonkey platform (www.surveymonkey.com) and comprised the following sections: (A) socio-demographic information, (B) previous losses, personal and family history of psychological disorders, (C) birth expectations before and after COVID-19, (D) concerns regarding pandemic consequences, (E) postpartum health and infant feeding, (F) perception of media and health professionals' information and communication on COVID-19, (G) psychometric evaluation: State Trait Anxiety Inventory (STAI-Y1, STAI-Y2), Symptom Checklist 90 (SCL-90), National Stressful Events Survey PTSD Short Scale (NSESSS).

Women's concerns were examined using a Likert scale (from 0 “not at all concerned” to 3 “very concerned”) regarding six issues: (I) their own health, (II) baby's health, (III) partner's health, (IV) elderly relatives' health, (V) baby's future and (VI) future of society.

Section E of the survey included 23 specific questions (Likert 0–3) investigating different domains regarding infant feeding with the purpose of assessing characteristics and burden of breastfeeding during the pandemic. It was developed by CR and AV and was derived from a series of open questions that were previously asked to women and then enclosed in Section E of COVID-ASSESS, called NECTAR (Newborn FEeding in emergenCy quesTionnAiRe). Methodological details, full text of the survey and raw data have been published (18).

This paper is a secondary analysis of the national survey COVID-ASSESS that included 2,448 women, of whom 1,307 were pregnant and 1,141 postpartum. Participants were considered eligible to be included in this *post-hoc* analysis if (a) they were in the postpartum period; (b) their babies were younger than 6 months; they were (c1) exclusively breastfeeding since birth at the time of the interview, or (c2) they were feeding their children with formula or mixed-feeding at the time of the interview; (d) they had never fed their children with any solid food. Infant feeding at the time of interview was reported according to the following categories: (1) exclusive breastfeeding since birth (defined as breastfeeding with no other food or drink), (2) mixed feeding (children were currently fed infant formula in addition to breast milk), (3) formula feeding (children were currently fed only with formula).

### Statistical analysis and data presentation

Survey responses were downloaded and extracted from the online survey tool, Surveymonkey, and imported into Excel for data management. Data were cleaned and checked. Quantitative data were imported into Stata BE 17.0 (StataCorp) for statistical analysis. Responses were analyzed for all women and segregated based on during or post-lockdown, infant feeding and COVID-19 concerns.

Descriptive statistics were used to analyze quantitative data. Categorical data were reported as frequencies and percentages and compared using the chi-squared test, whereas continuous data were reported as mean values with standard deviations and compared using *t*-test, if normally distributed. All results were considered to be statistically significant at *p* < 0.05.

All NECTAR items were scored 0–3 (“I fully disagree” to “I fully agree”), scores of some items were then reversed (3-0), so that higher NECTAR mean scores indicated better adjustment and positive attitude with infant feeding.

Three multivariate analyses were performed. The first was conducted to evaluate the association between COVID-19 concerns and psychopathological symptoms and the following variables: (a) maternal age, (b) first pregnancy or multiparity, (c) history of previous losses, (d) assisted reproduction, (e) child's age, (f) family history of psychiatric diseases [anxiety, depression, bipolar disorder, obsessive-compulsive disorder (OCD), eating disorders (ED), others], (g) history of psychiatric diagnoses (anxiety, depression, bipolar disorder, OCD, ED, other), (h) lockdown duration, (i) feeding pattern.

The second analysis aimed to investigate the association between the possibility that a woman was not exclusively breastfeeding with the variables mentioned above from (a) to (h), plus (l) current state anxiety (SCL90-Anx, STAI-Y1 e STAI-Y2).

The third analysis included in the model those factors able to independently predict the risk of not exclusively breastfeeding and was used to draw a nomogram calculating the risk.

Responders' location, feeding practices and concerns about the COVID-19 pandemic were mapped by regional areas across Italy using Tableau Desktop 2021.3 (Tableau Software, LLC).

## Findings

### Sample characteristics

**Figure 1:**
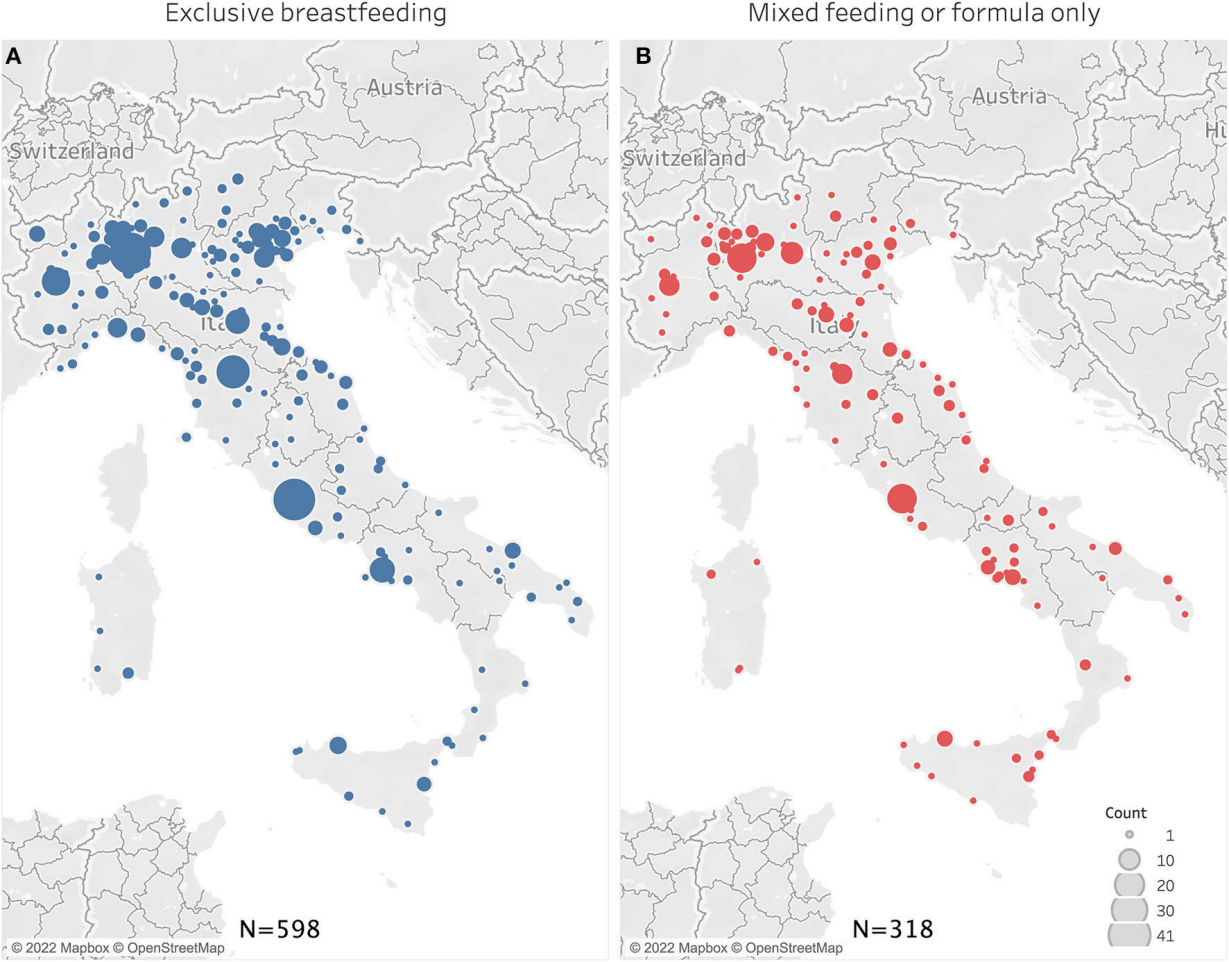
Geographical distribution of women who reported exclusive breastfeeding **(A)** or formula/mixed feeding **(B)**.

A total of 914 women satisfied inclusion criteria for this *post-hoc* analysis. Socio-demographic characteristics according to infant feeding are reported in Table 1. No significant differences were present between groups regarding maternal age and education, duration of lockdown (number of days women were confined home) and geographical distribution (Table 1, Figure 1); 551 women were living in Northern Italy, 195 in Central Italy and 168 in Southern Italy.

**Table 1:** Characteristics of the sample.

	**Group**									
	**Excl breastfeeding**		**Mixed**		**Formula only**		**Total**		**χ2**	***p***
	**No.**	**%**	**No.**	**%**	**No.**	**%**	**No.**	**%**		
**Age classes**										
18–25	16	2.7%	1	0.2%	1	0.2%	18	1.97%	9.929	0.128
25–30	67	11.2%	21	3.5%	15	2.5%	103	11.27%		
30–35	258	43.1%	77	12.9%	40	6.7%	375	41.03%		
>35	257	43.0%	105	17.6%	56	9.4%	418	45.73%		
**Level of education**										
Secondary	189	31.6%	66	11.0%	46	7.7%	301	32.93%	8.666	0.070
Post-secondary non-tertiary	135	22.6%	44	7.4%	31	5.2%	210	22.98%		
Tertiary	274	45.8%	94	15.7%	35	5.9%	403	44.09%		
**Baby age (0–6 m)**										
<1.5	234	39.1%	49	8.2%	38	6.4%	321	35.12%	15.914	0.003
1.5–4	185	30.9%	77	12.9%	34	5.7%	296	32.39%		
>4	179	29.9%	78	13.0%	40	6.7%	297	32.49%		
**Days since lockdown**										
<15	205	34.3%	73	12.2%	45	7.5%	323	35.34%	2.090	0.719
15–30	179	29.9%	62	10.4%	34	5.7%	275	30.09%		
>30	214	35.8%	69	11.5%	33	5.5%	316	34.57%		
**Total**	598	100.0%	204	100.0%	112	100.0%	914	100.0%		

**Figure 2:**
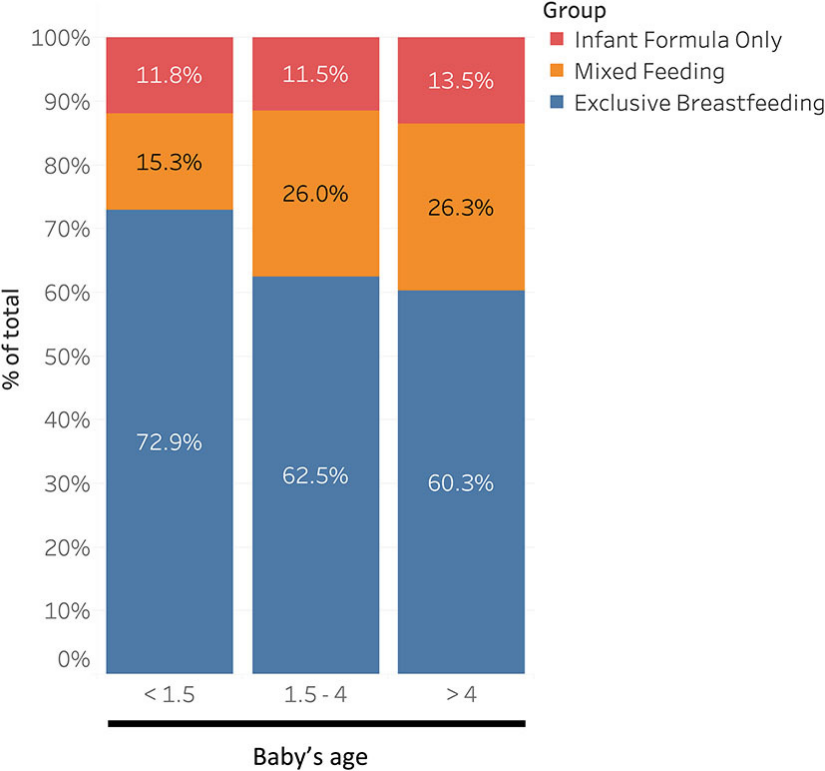
Distribution of feeding practices according to baby's age.

Regarding infant age, babies in the first age tertile (<1.5 months) were more likely to be exclusively breastfed (72.9%) than those in the second age tertile (1.5–4 months; 62.5%) and in the third (>4 months; 60.3%, χ^2^ = 15.9, *p* < 0.01) (Figure 2).

### Women's concerns and newborn feeding practices

**Figure 3:**
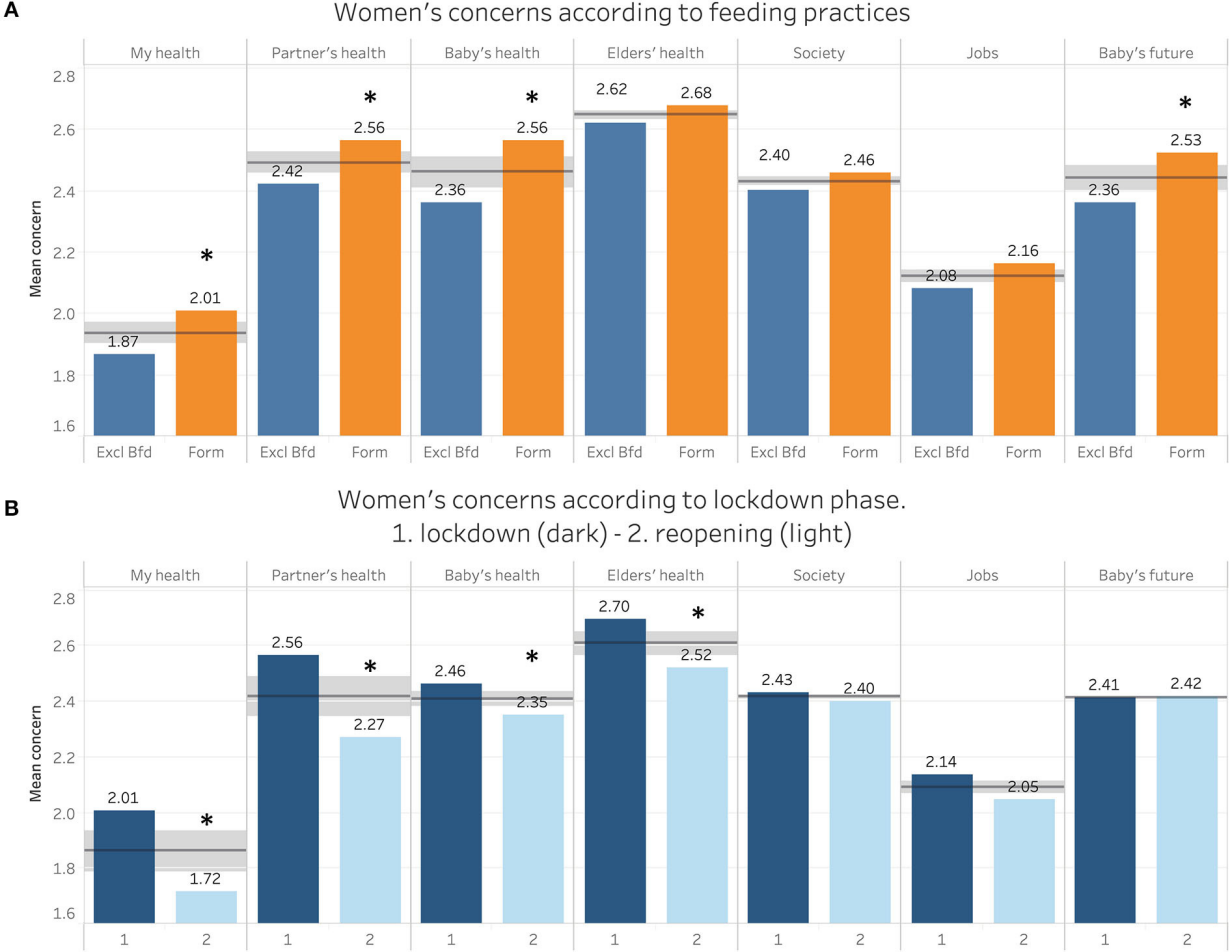
Concerns of women regarding several health and social domains, according to feeding practices **(A)** and pandemic phase **(B)**. Numbers represent median values, horizontal line represent median, shaded area represents quartiles, *p < 0.05.

Table 2 and Figure 3 report women's concerns about COVID-19. Women whose babies were exclusively breastfed were generally less concerned than other women regarding the pandemic (overall concern 2.42 ± 0.5 vs. 2.30 ± 0.5; *p* < 0.01) with significant differences regarding their personal health, their partner's health, baby's health and baby's future (Figure 3A).

**Table 2:** Concerns on health and social issues according to feeding practices.

	**Feeding pattern**							
	**Breastfeeding**		**Formula or mixed**		**Total**		**χ^2^**	***p***
	**No.**	**%**	**No.**	**%**	**No.**	**%**		
**Concerns on personal health**								
None	24	5.3%	8	3.7%	32	4.8%	4.052	0.256
Low	134	29.8%	55	25.6%	189	28.4%		
Medium	170	37.8%	79	36.7%	249	37.4%		
High	122	27.1%	73	34.0%	195	29.3%		
**Concerns on partner's health**								
None	3	0.7%	2	0.9%	5	0.8%	7.327	0.062
Low	48	10.7%	13	6.0%	61	9.2%		
Medium	154	34.3%	62	28.8%	216	32.5%		
High	244	54.3%	138	64.2%	382	57.5%		
**Concerns on baby's health**								
None	5	1.1%	1	0.5%	6	0.9%	10.018	0.018
Low	78	17.3%	23	10.7%	101	15.2%		
Medium	117	25.9%	45	20.9%	162	24.3%		
High	251	55.7%	146	67.9%	397	59.6%		
**Concerns on elders' health**								
None	8	1.8%	1	0.5%	9	1.4%	3.253	0.354
Low	19	4.2%	11	5.1%	30	4.5%		
Medium	109	24.2%	44	20.6%	153	23.0%		
High	315	69.8%	158	73.8%	473	71.1%		
**Concerns on society**								
None	3	0.7%	1	0.5%	4	0.6%	1.267	0.737
Low	25	5.5%	9	4.2%	34	5.1%		
Medium	210	46.6%	95	44.2%	305	45.8%		
High	213	47.2%	110	51.2%	323	48.5%		
**Concerns on jobs**								
None	13	2.9%	10	4.7%	23	3.5%	7.152	0.067
Low	103	22.8%	43	20.0%	146	21.9%		
Medium	169	37.5%	64	29.8%	233	35.0%		
High	166	36.8%	98	45.6%	264	39.6%		
**Concerns on baby's future**								
None	11	2.4%	3	1.4%	14	2.1%	7.901	0.048
Low	53	11.8%	19	8.8%	72	10.8%		
Medium	149	33.0%	55	25.6%	204	30.6%		
High	238	52.8%	138	64.2%	376	56.5%		
**Total**	451	100.0%	215	100.0%	666	100.0%		

A significant decrease between lockdown and reopening was observed in all health-related concerns while concerns regarding future did not decrease significantly (Figure 3B).

### Psychological distress and newborn feeding practices

Self-reported history of anxiety (35.4 vs. 26.4%, χ2 = 8.1, *p* < 0.01) and trait anxiety (STAI-Y2 42.7 ± 10.4 vs. 40.3 ± 9.3; *p* < 0.01) were more frequent in mothers not exclusively breastfeeding, while other psychopathological diagnosis did not significantly differ between groups. There was no evidence of higher prevalence of family history of mental disorders in either group.

Women whose babies were exclusively breastfed showed lower scores of general psychopathological distress (SCL90_GSI: 0.77 ± 0.5 vs. 0.89 ± 0.6; *p* < 0.05) and in several psychopathological domains such as state anxiety (STAI-Y1: 53.7 ± 12.2 vs. 56.7 ± 12.0; SCL90_ANX 0.82 ± 0.6 vs. 0.94 ± 0.7; *p* < 0.05), somatization (SCL90_Som: 0.74 ± 0.6 vs. 0.95 ± 0.7; *p* < 0.01) and PTSD (NSESSS: 13.6 ± 7.8 vs. 15.4 ±7.9; *p* < 0.01).

**Figure 4:**
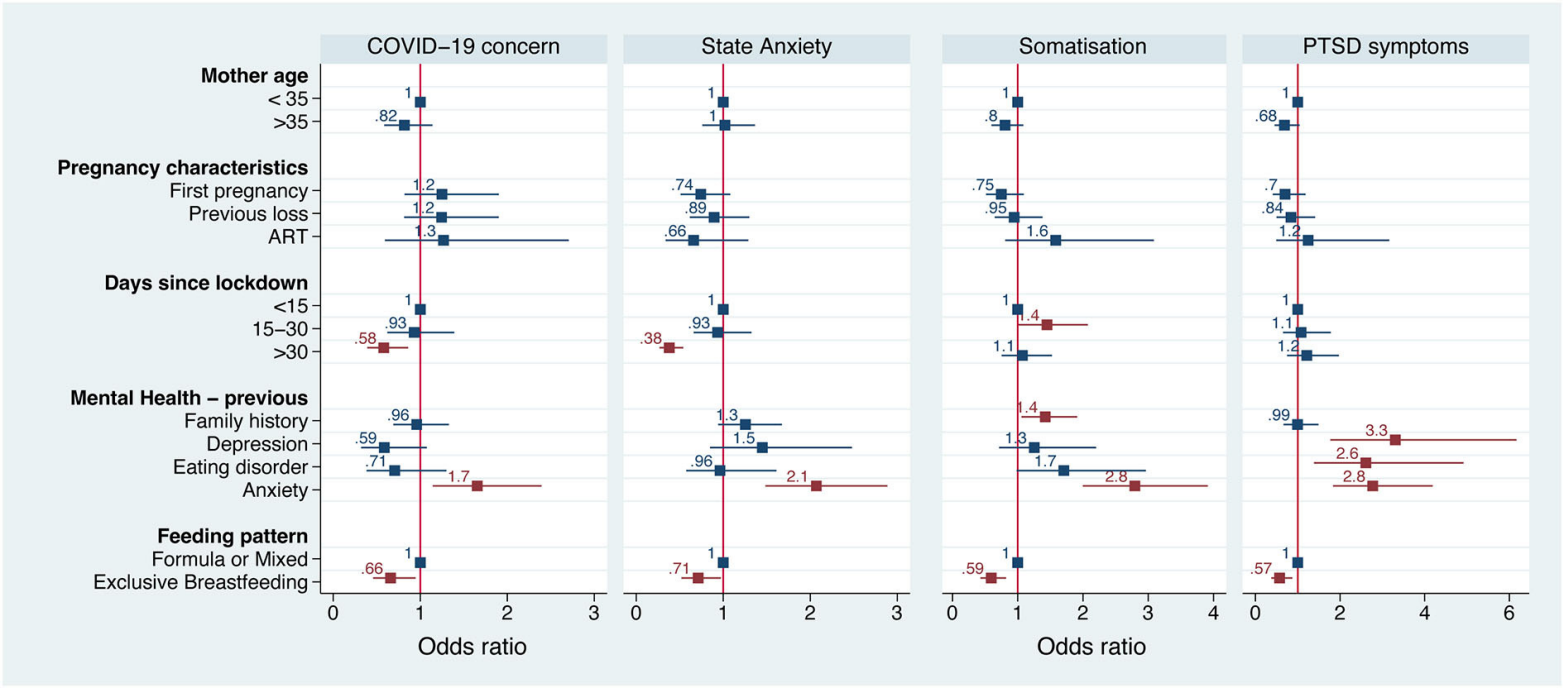
Forest plot of the logistic regression of several psychopathological parameters according to the most theoretically and statistically significant variables. Numbers and squares represent odds ratios, horizontal line represents 95% CI, red color *p* < 0.05.

The first multivariate analysis showed that COVID-19 concern, state anxiety, somatization and PTSD symptoms were associated with a number of independent factors, among which a previous psychological history of anxiety (risk factor) and exclusive breastfeeding (protective factor) were the only ones that affected all studied parameters (Figure 4). After a month of lockdown, anxiety and COVID-19 concern tended to improve, while PTSD symptoms remained unchanged. All coefficients of the multivariate analysis are reported in Supplementary Table 1.

### Newborn feeding attitudes during the pandemic

**Figure 5:**
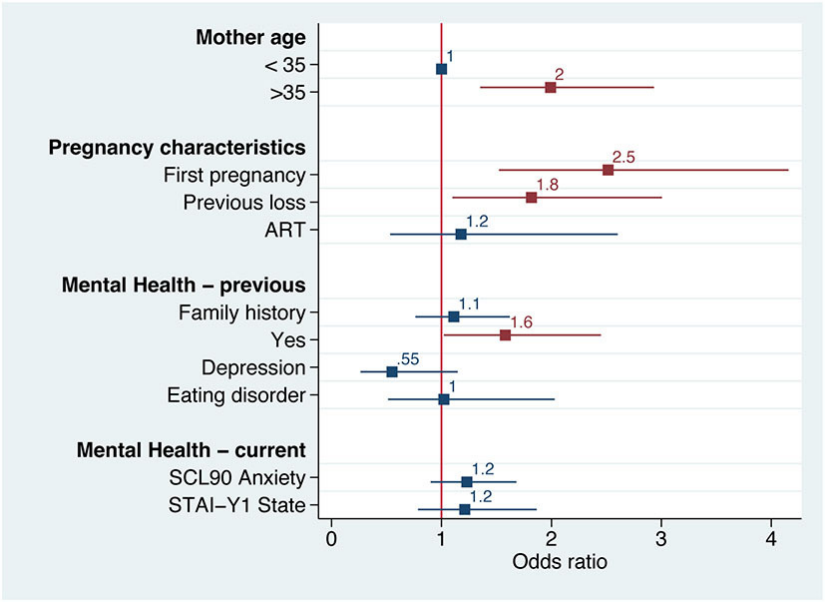
Forest plot of the logistic regression of probability of not exclusively breastfeeding according to the most theoretically and statistically significant variables. Numbers and squares represent odds ratios, horizontal line represents 95% CI, red color *p* < 0.05.

In the second multivariate analysis, four medical and socio-demographic factors, independent of each other, were associated with the possibility that a woman fed her infant with formula during the pandemic: age >35 [OR 1.99 (1.35–2.93)], primiparity [OR 2.52 (1.52–4.15)], previous perinatal losses [OR 1.82 (1.09–3.00)] and previous history of anxiety [OR 1.58 (1.01–2.44)] (Figure 5; Supplementary Table 2).

NECTAR items that scored significantly differently between the exclusively breastfeeding group and the other group are reported in Table 3 (higher scores reflect a positive evaluation of their feeding experience by mothers). NECTAR mean score was higher in exclusively breastfeeding women compared to non-exclusively breastfeeding women (respectively 1.91 ± 0.3 vs. 1.64 ± 0.4; *p* < 0.0001).

**Table 3:** Mean scores of the Newborn FEeding in emergenCy quesTionnAiRe (NECTAR) items.

**Item**	**Breastfeeding** ***(Mean ±SD)***	**Formula or mixed** ***(Mean ±SD)***	***p***
1. Feeding is continuing as regularly as before the emergency	2.43 ± 0.75	1.67 ± 1.08	<0.0001
2. Feeding has been modified (increased) respect before the emergency^*^	2.52 ± 0.78	2.42 ± 0.84	0.0828
3. Feeding has been modified (reduced) respect before the emergency^*^	2.81 ± 0.50	2.50 ± 0.87	<0.0001
4. Feeding has become very tiring for me^*^	2.39 ± 0.77	1.85 ± 1.02	<0.0001
5. Feeding has become very tiring during the night^*^	1.94 ± 0.92	1.74 ± 1.02	0.0041
6. I am considering changing feeding mode (ex. adding some formula)^*^	2.66 ± 0.69	1.37 ± 1.15	<0.0001
7. I have considered anticipating weaning^*^	2.72 ± 0.64	2.56 ± 0.77	0.0008
8. Feeding in these days is bothering me^*^	2.73 ± 0.55	2.50 ± 0.82	<0.0001
9. Feeding is the only thing I can do with my baby, as before the emergency	1.61 ± 1.06	1.22 ± 1.06	<0.0001
10. I'm afraid to transmit my tension to my baby through the milk^*^	1.89 ± 1.01	1.90 ± 1.14	0.909
11. When feeding my baby, I feel at peace	2.26 ± 0.68	1.90 ± 0.90	<0.0001
12. When feeding my baby, I can forget (not think to) the emergency	1.81 ± 0.94	1.60 ± 1.01	0.002
13. Feeding my baby, I keep them safe	2.53 ± 0.64	2.08 ± 0.86	<0.0001
14. Coming back home with my baby was as I had imagined it	1.35 ± 1.02	1.06 ± 1.05	0.0001
15. Having a satisfactory routine is now complicated^*^	1.18 ± 0.87	1.19 ± 0.95	0.9223
16. Not being able to go out with the baby annoys me^*^	0.91 ± 0.82	0.84 ± 0.86	0.2488
17. Not being able to receive visits from family and friends annoys me^*^	1.17 ± 0.95	1.12 ± 1.00	0.4467
18. Time never passes^*^	1.81 ± 0.95	1.73 ± 1.06	0.2637
19. I'd like to be more carefree^*^	0.74 ± 0.81	0.63 ± 0.81	0.0631
20. I would like to enjoy my baby more^*^	1.31 ± 1.06	1.11 ± 1.07	0.0087
21. Staying home with my baby and my partner gives me security	2.30 ± 0.72	2.33 ± 0.72	0.5733
22. I can rest as I would like and recover enough energy	1.38 ± 0.82	1.20 ± 0.85	0.0026
23. Concern about the emergency absorbs a lot of my energy^*^	1.49 ± 0.79	1.29 ± 0.88	0.0009

All items were scored 0–3 (“I fully disagree” → “I fully agree”), scores of items marked with

* were then inverted (3-0), so that higher scores indicated better adjustment and more positive attitude with baby's feeding.

**Figure 6:**
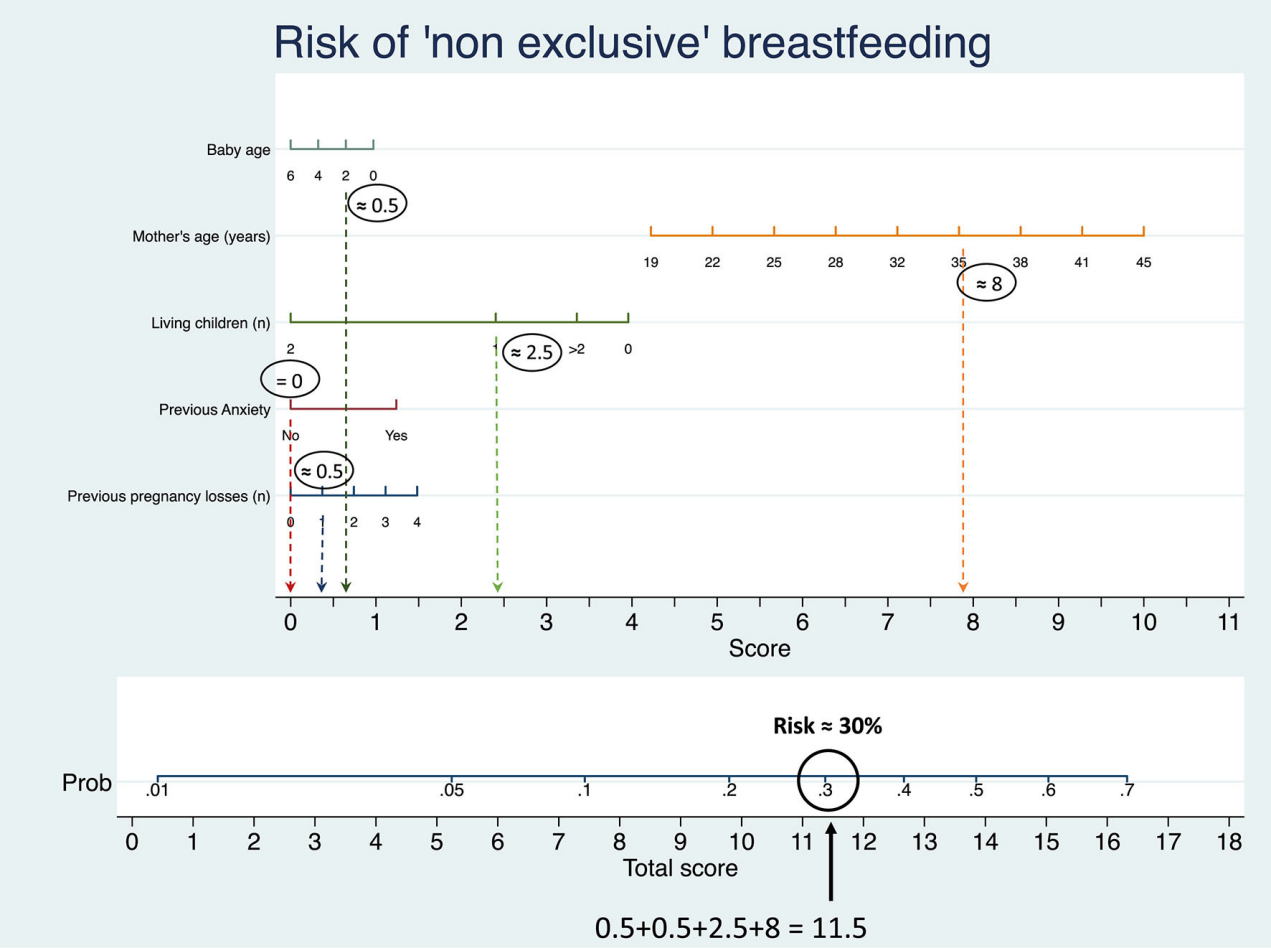
Nomogram derived by logistic regression of probability of not exclusively breastfeeding according to baby's age, mother's age, number of living children, presence of previous anxiety and number of previous losses.

Using a third multivariate analysis, we drew a nomogram to calculate the risk of not exclusively breastfeeding based on women's demographic and medical history factors (Figure 6; Supplementary Figure 1). Figure 6 presents an example of how to read the diagram: consider the case of a 35 year old woman, whose baby is 2 months old, with another child, no previous anxiety history and a past miscarriage. Finding intercepts for each value on the x axis we can calculate the score, that in this case is 12.5. On the probability scale at the bottom of the figure, a score of 12.5 corresponds to a risk of not exclusively breastfeeding of ≈30%. Higher values correspond to higher risks of using formula or mixed feeding. A clean nomogram is provided as Supplementary material.

**Figure 7:**
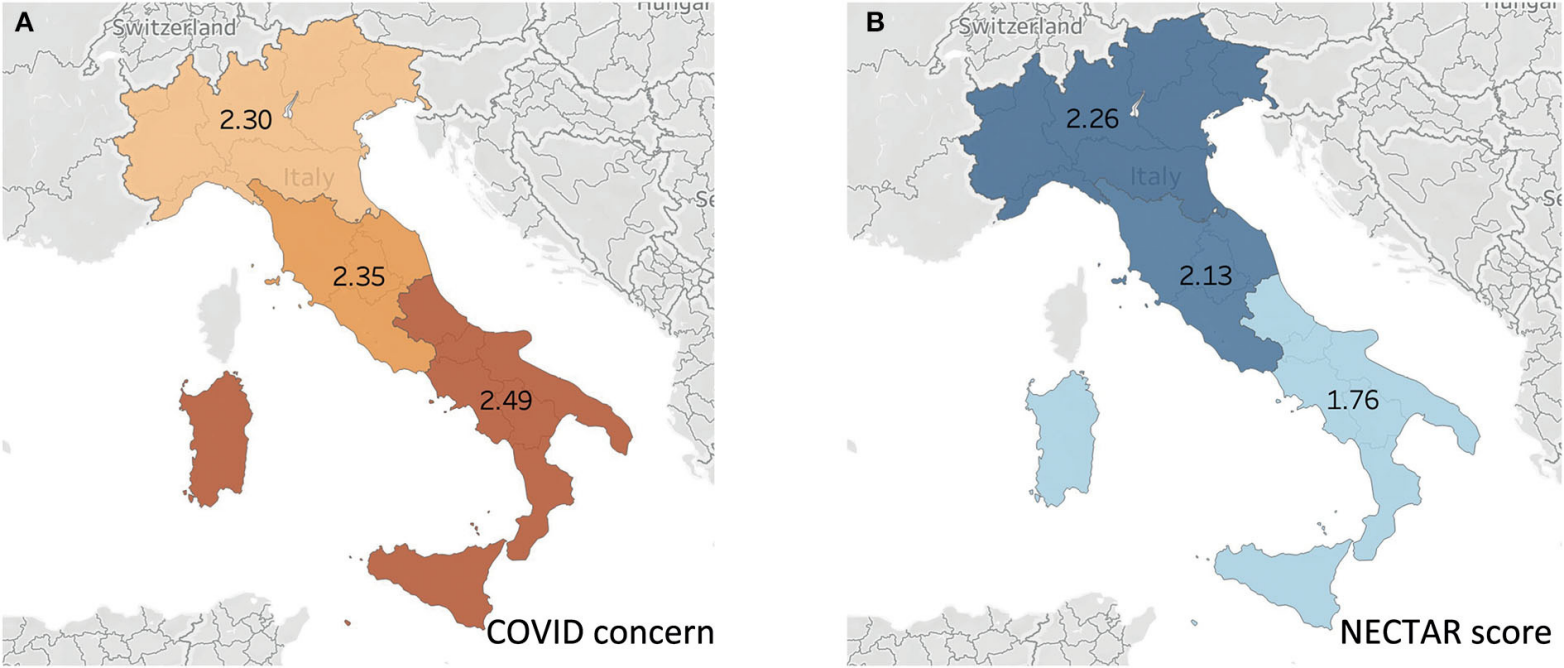
Geographical distribution of COVID-19 concern **(A)** and NECTAR score **(B)** in different Italian zones.

Both COVID concern and NECTAR mean scores were differently distributed between northern, central and southern Italy (Figure 7).

## Discussion

To the best of our knowledge, this is the first study that investigates maternal mental health status and infant feeding methods in two phases of the COVID-19 pandemic.

Postpartum women's concerns about COVID-19 were relevant and comparable to those of pregnant women (19, 20), nevertheless the scores significantly decreased between phase 1 (lockdown period) and phase 2 (reopening). Although this might be expected, this is the first time that such a decrease is described, since previous research from the overarching study focused on COVID-19 and women's mental health in Italy during phase 1. We found a reduction in anxiety and concerns on general health issues except for “baby's health,” while the fear for the impact of the pandemic on society did not diminish. In our opinion, concerns regarding general health diminished due to the more widespread knowledge that COVID-19 generally had a benign course in the young. This hypothesis is supported by an Italian paper that showed after the first wave a decrease in worry and a feeling of better control of viral transmission in the general population (21).

Women, whose infants were not exclusively breastfed were generally more concerned about all aspects of their own and their baby's health compared to women exclusively breastfeeding. This effect might have been a proxy of anxiety and not an actual protective effect of breastfeeding, for example women who were already more anxious might have used infant formula. Indeed, there is evidence that anxiety during the postpartum period is negatively associated with breastfeeding exclusivity and duration (22, 23). Furthermore, women with underlying mental health disorders tend to stop breastfeeding earlier, within a month after birth, suggesting the need to identify this population before childbirth to provide enhanced support for this cohort during lactation (24).

We have already shown that in pregnant women a history of anxiety was the greatest predictor of concern for the pandemic and childbirth (19, 20). In this secondary analysis, we show the same association in mothers not exclusively breastfeeding. While there was no evidence of higher prevalence of trait anxiety in the exclusively breastfeeding group, a multivariate analysis showed that being a woman over 35 years with a history of anxiety, primiparity or being pregnant after a loss, all were associated with an increased use of infant formula. As such this group of women could require greater advice and support on breastfeeding during current or future epidemics, and other public health crises.

On the other hand, in the first multivariate analysis conducted, exclusive breastfeeding seems to be a protective factor, independent of psychopathological history, for the development of symptoms of stress, anxiety, somatization and general concern for the pandemic. It should be taken into account that breastfeeding issues (e.g., concerns with adequacy of milk supplies, nipple pain or mastitis) are linked to poorer maternal mood (25). In our opinion, these data strongly support an action by the Italian National Health Care Service to improve and prioritize pregnancy and birth care, with both specific attention to the promotion of exclusive breastfeeding and an adequate breastfeeding education, in order to achieve a better mental health status of mothers (14–16).

Our results show how in women who remained in lockdown for over a month, anxiety and concerns were lower, while no statistically significant change was observed in PTSD symptoms. These results could be explained by hypothesizing that more information about COVID-19 and a sense of better pandemic control was able to decrease anxiety and worry. In fact, during the time of this study, a lot more information was circulating about the health impacts of COVID-19 in pregnant women (21). Further studies are needed to confirm or dispel these hypotheses.

Answers to the NECTAR questionnaire and mean score were very different between breastfeeding and formula groups. Our data suggest that women who were exclusively breastfeeding showed higher adaptability toward pandemic challenges and greater self-confidence, as well as lower levels of psycho-physical fatigue and difficulty coping. Therefore, strategies aimed at increasing women's self-confidence could be useful in increasing breastfeeding rates in public health emergencies. This is particularly true in the first few months, exclusively breastfeeding was more frequent in mothers with babies under 1.5 months than in older ones (Figure 2), in keeping with European trend (26).

Moreover, women's ability to adapt to the health emergency and to experience feeding their infant in a positive way showed important geographical differences. In the South concerns about COVID-19 were higher than in the rest of the country and the NECTAR index score was lower. It should be taken into account that at the time of data collection there was no health emergency related to COVID-19 in the South, whereas Northern Italy was having the highest global rate of COVID-19 confirmed cases as well as the highest mortality rate in the world (19). It could be also possible that the pandemic did not directly impact breastfeeding rates, but concerns about the pandemic were influenced by a pre-existing situation, for example, women who had a familiar history of anxiety tended to use formula more frequently. We have two hypotheses to explain these results.

The first one is that those who respond to acute stress with anxious coping could find breastfeeding more difficult. We previously underlined the link between anxiety during the postpartum period and breastfeeding (22, 23). However, there is no specific evidence regarding the connection between antenatal psychological difficulties (those related to pandemic emergency), the relative coping strategies, and difficulties with breastfeeding. If this association should be confirmed, it could be useful to identify women with anxious coping and support them before and after birth (27, 28). For this reason, we have drawn a nomogram (Figure 6) based on demographic and medical factors significantly associated with not-exclusively breastfeeding, so that women at higher risk of feeding difficulties can be identified.

The second hypothesis is neurobiological: we have shown that women who are going through the stress of primiparity, or have a history of anxiety, or previous losses tend to breastfeed less; this deprives them of the beneficial mental health effects of lactation, further increasing their psychological difficulties. For example, it has been shown that the hormone oxytocin, that is strongly released during breastfeeding, improves physiological and psychological adaptation in mothers reducing cortisol, anxiety and increasing prolactin levels (29).

We would also like to comment regarding the role of fathers and social support network, two factors of great importance for most breastfeeding women. An appropriate support network makes in fact mothers and babies able to start, find and maintain their own breastfeeding pattern, with the best satisfaction for both. The role of fathers in supporting exclusive breastfeeding has been widely investigated: a recent review showed that partner support is essential for infant feeding and can influence new mothers' decision to initiate, continue or cease breastfeeding (30). Verbal encouragement to new mothers and other behaviors such as sensitivity of the partner to the nursing mother's needs, assistance in preventing and managing breastfeeding difficulties, helping with household and childcare tasks such as bathing, playing, and singing with the baby play a pivotal role in supporting breastfeeding (30, 31). Partner's positive attitude toward breastfeeding is also significantly associated with the increased likelihood of the infant being breastfed (32). Unfortunately, in this survey we are not able to address the role of fathers in breastfeeding during COVID-19 lockdown in Italy, since the instrument used lacked a specific section on the subject (18).

Finally, regarding professional support, we have previously highlighted the psychosocial impact of changes in antenatal care and birth care that pandemic and restrictive measures caused in Italy (19, 20). Especially during the first lockdown, the lack of information and the change in maternity units' organization had a strong impact on pregnant women: many of them were left without information for weeks and, due to restrictive measures, part of respectful care practices for a positive childbirth experience were simply not provided (19, 33). Even though in the present survey we did not specifically address the point, it is reasonable to hypothesize that lack of the presence of the companion of choice for labor and childbirth, decreased possibility of skin-to-skin contact and rapid discharge without a proper in person follow up, may have played a role in initiating and maintaining breastfeeding (34).

## Strength and limitation

While exclusive breastfeeding was clearly defined in the original survey (breastfeeding with no other food or drink, not even water) and the same can be said for infant formula feeding (children were fed only with formula), no specific information was requested regarding the amount of formula used in the “mixed feeding” group. The survey was distributed through a charity dealing with perinatal loss support. Thus, the sample is more likely to have experienced a perinatal loss than the general population. Although having had previous losses was associated with higher use of infant formula, the multivariate analysis demonstrated that breastfeeding is the only independent factor that influences COVID-19 concerns and mental health status. So, the difference of our sample compared to the general population could already have been self-corrected.

## Conclusions

This paper is a *post-hoc* analysis of the COVID-ASSESS study and infant feeding was not the primary focus. We examined the mental health status of women during the first wave of the COVID-19 pandemic in Italy. We found that breastfeeding during the pandemic appeared to be an independent factor associated with reduced concern for COVID-19, fewer and less intense psychopathological symptoms, and a more positive experience of infant feeding. Systems should be in place to support all women to achieve their breastfeeding goals, with special attention to primiparous women over 35 years, and those who have experienced previous pregnancy losses or a history of anxiety. Finally, in Italy more resources should be invested to provide an enabling environment for breastfeeding and make breastfeeding easier for women in all regions, especially Central and Southern regions of Italy.

## Data availability statement

The datasets presented in this study can be found in online repositories. The names of the repository/repositories and accession number(s) can be found at: https://data.mendeley.com/datasets/cn38pbwn7r/1.

## Ethics statement

The studies involving human participants were reviewed and approved by Human Research ethical approval to conduct the survey was received from Florence University Ethics Committee (Prot. n. 006897). Each participant gave their explicit consent in an online form before enrolment. Written informed consent for participation was not required for this study in accordance with the national legislation and the institutional requirements.

## Author contributions

CR and AV led this research including proposal write up and designed the instrument. CR, AV, RB, and VR collected and analyzed data. CR, AW, VR, LA, LM, and AV discussed data and wrote the manuscript. All authors read and approved the final manuscript.

## Funding

CiaoLapo Foundation for Healthy Pregnancy and Perinatal Loss Support provided infrastructure for conducting the study (documents, questionnaires, material, software, web platforms, and open access, etc).

## Conflict of interest

The authors declare that the research was conducted in the absence of any commercial or financial relationships that could be construed as a potential conflict of interest.

## Publisher's note

All claims expressed in this article are solely those of the authors and do not necessarily represent those of their affiliated organizations, or those of the publisher, the editors and the reviewers. Any product that may be evaluated in this article, or claim that may be made by its manufacturer, is not guaranteed or endorsed by the publisher.
