# Supplementary material for: Exclusive breastfeeding and women's psychological well-being during the first wave of COVID-19 pandemic in Italy
Source: Front Public Health. 2022 Aug 23;10:965306. doi: 10.3389/fpubh.2022.965306 (PMC9445494; doi:10.3389/fpubh.2022.965306)
Supplement: Supplementary file 1 [file Table_1.DOCX]

|  | **COVID-19 concern** | | | **Anxiety** | | | **Somatization** | | | **PTSD symptoms** | | |
| --- | --- | --- | --- | --- | --- | --- | --- | --- | --- | --- | --- | --- |
|  | OR | 95% CI | *p* | OR | 95% CI | *p* | OR | 95% CI | *p* | OR | 95% CI | *p* |
| **Sociodemographic variables** |  |  |  |  |  |  |  |  |  |  |  |  |
| Age >35 | 0.82 | [0.58-1.14] | 0.236 | 1.02 | [0.75-1.36] | *0.902* | 0.80 | [0.59-1.08] | *0.156* | 0.68 | [0.44-1.04] | *0.077* |
|  |  |  |  |  |  |  |  |  |  |  |  |  |
| First pregnancy | 1.25 | [0.82-1.90] | 0.299 | 0.74 | [0.50-1.07] | *0.119* | 0.75 | [0.51-1.09] | *0.133* | 0.70 | [0.41-1.18] | *0.183* |
|  |  |  |  |  |  |  |  |  |  |  |  |  |
| Previous loss | 1.25 | [0.81-1.90] | 0.310 | 0.89 | [0.61-1.29] | *0.559* | 0.95 | [0.64-1.38] | *0.770* | 0.84 | [0.49-1.40] | *0.507* |
|  |  |  |  |  |  |  |  |  |  |  |  |  |
| Number of losses | 0.72 | [0.30-1.72] | 0.458 | 1.55 | [0.76-3.14] | *0.220* | 1.09 | [0.44-2.63] | *0.855* | 0.98 | [0.35-2.71] | *0.975* |
|  |  |  |  |  |  |  |  |  |  |  |  |  |
| Assisted reproductive technology | 1.27 | [0.59-2.70] | 0.541 | 0.66 | [0.33-1.28] | *0.223* | 1.58 | [0.80-3.08] | *0.180* | 1.25 | [0.49-3.16] | *0.644* |
|  |  |  |  |  |  |  |  |  |  |  |  |  |
| Baby’s age | 0.90 | [0.80-1.01] | 0.085 | 1.01 | [0.91-1.11] | *0.861* | 1.13 | [0.99-1.25] | *0.060* | 1.03 | [0.88-1.18] | *0.706* |
|  |  |  |  |  |  |  |  |  |  |  |  |  |
| Lockdown 15-30 days | 0.93 | [0.62-1.39] | 0.723 | 0.93 | [0.65-1.32] | *0.703* | 1.45 | [0.99-2.07] | *0.063* | 1.08 | [0.65-1.77] | *0.769* |
|  |  |  |  |  |  |  |  |  |  |  |  |  |
| Lockdown >30 days | 0.58 | [0.39-0.86] | 0.007 | 0.38 | [0.26-0.54] | *<0.001* | 1.07 | [0.75-1.52] | *0.697* | 1.21 | [0.74-1.97] | *0.436* |
| **Mental health (previous)** |  |  |  |  |  |  |  |  |  |  |  |  |
| Family psychological history | 0.96 | [0.69-1.32] | *0.800* | 1.26 | [0.94-1.67] | *0.122* | 1.42 | [1.05-1.91] | *0.020* | 0.99 | [0.66-1.48] | *0.976* |
|  |  |  |  |  |  |  |  |  |  |  |  |  |
| Self-reported depression | 0.59 | [0.31-1.07] | *0.084* | 1.45 | [0.84-2.47] | *0.174* | 1.25 | [0.71-2.20] | *0.432* | 3.31 | [1.77-6.16] | *<0.001* |
|  |  |  |  |  |  |  |  |  |  |  |  |  |
| Self-reported eating disorder | 0.71 | [0.38-1.29] | *0.264* | 0.96 | [0.57-1.61] | *0.885* | 1.71 | [0.98-2.96] | *0.058* | 2.61 | [1.38-4.92] | *0.003* |
|  |  |  |  |  |  |  |  |  |  |  |  |  |
| Self-reported OCD | 0.47 | [0.10-2.04] | *0.314* | 1.34 | [0.33-5.35] | *0.679* | 0.89 | [0.24-3.20] | *0.859* | 0.62 | [0.10-3.83] | *0.610* |
|  |  |  |  |  |  |  |  |  |  |  |  |  |
| Self-reported anxiety | 1.65 | [1.14-2.39] | *0.008* | 2.07 | [1.48-2.88] | *<0.001* | 2.79 | [1.99-3.91] | *<0.001* | 2.77 | [1.83-4.18] | *<0.001* |
| **Exclusive breastfeeding** | 0.66 | [0.45-0.94] | *0.023* | 0.71 | [0.51-0.97] | *0.033* | 0.59 | [0.42-0.82] | *0.002* | 0.57 | [0.37-0.87] | *0.010* |

**Supplementary table 1.** Coefficients of the first logistic regression for COVID-19 concern, state anxiety, somatization, and PTSD symptoms.
