# Supplementary material for: Exclusive breastfeeding and women's psychological well-being during the first wave of COVID-19 pandemic in Italy
Source: Front Public Health. 2022 Aug 23;10:965306. doi: 10.3389/fpubh.2022.965306 (PMC9445494; doi:10.3389/fpubh.2022.965306)
Supplement: Supplementary file 2 [file Table_2.DOCX]

|  | **Risk of not exclusively breastfeeding** | | |
| --- | --- | --- | --- |
|  | Odds Ratio | 95% CI | *p* |
|  |  |  |  |
| **Sociodemographic variables** |  |  |  |
| Age >35 | 1.99 | [1.35-2.93] | *<0.001* |
|  |  |  |  |
| First pregnancy | 2.52 | [1.52-4.15] | *<0.001* |
|  |  |  |  |
| Previous loss | 1.82 | [1.09-3.00] | *0.020* |
|  |  |  |  |
| Assisted reproductive technology | 1.18 | [0.53-2.60] | *0.686* |
|  |  |  |  |
| Lockdown 15-30 days | 0.90 | [0.57-1.40] | *0.636* |
|  |  |  |  |
| Lockdown >30 days | 0.84 | [0.52-1.33] | *0.458* |
|  |  |  |  |
| **Mental health (previous)** |  |  |  |
| Family psychological history | 1.11 | [0.76-1.62] | *0.582* |
|  |  |  |  |
| Self reported anxiety | 1.58 | [1.01-2.44] | *0.041* |
|  |  |  |  |
| Self reported depression | 0.55 | [0.26-1.14] | *0.111* |
|  |  |  |  |
| Self reported eating disorder | 1.02 | [0.51-2.02] | *0.953* |
|  |  |  |  |
| Self reported OCD | 0.92 | [0.15-5.34] | *0.923* |
|  |  |  |  |
| **Mental health (current)** |  |  |  |
| SCL90 - Anxiety | 1.23 | [0.90-1.68] | *0.192* |
|  |  |  |  |
| STAI-Y1 | 1.21 | [0.78-1.86] | *0.384* |

**Supplementary table 2.** Coefficients of the logistic regression for risk of not exclusively breastfeeding.
