# Supplementary figures and images for: Exclusive breastfeeding and women's psychological well-being during the first wave of COVID-19 pandemic in Italy
Source: Front Public Health. 2022 Aug 23;10:965306. doi: 10.3389/fpubh.2022.965306 (PMC9445494; doi:10.3389/fpubh.2022.965306)

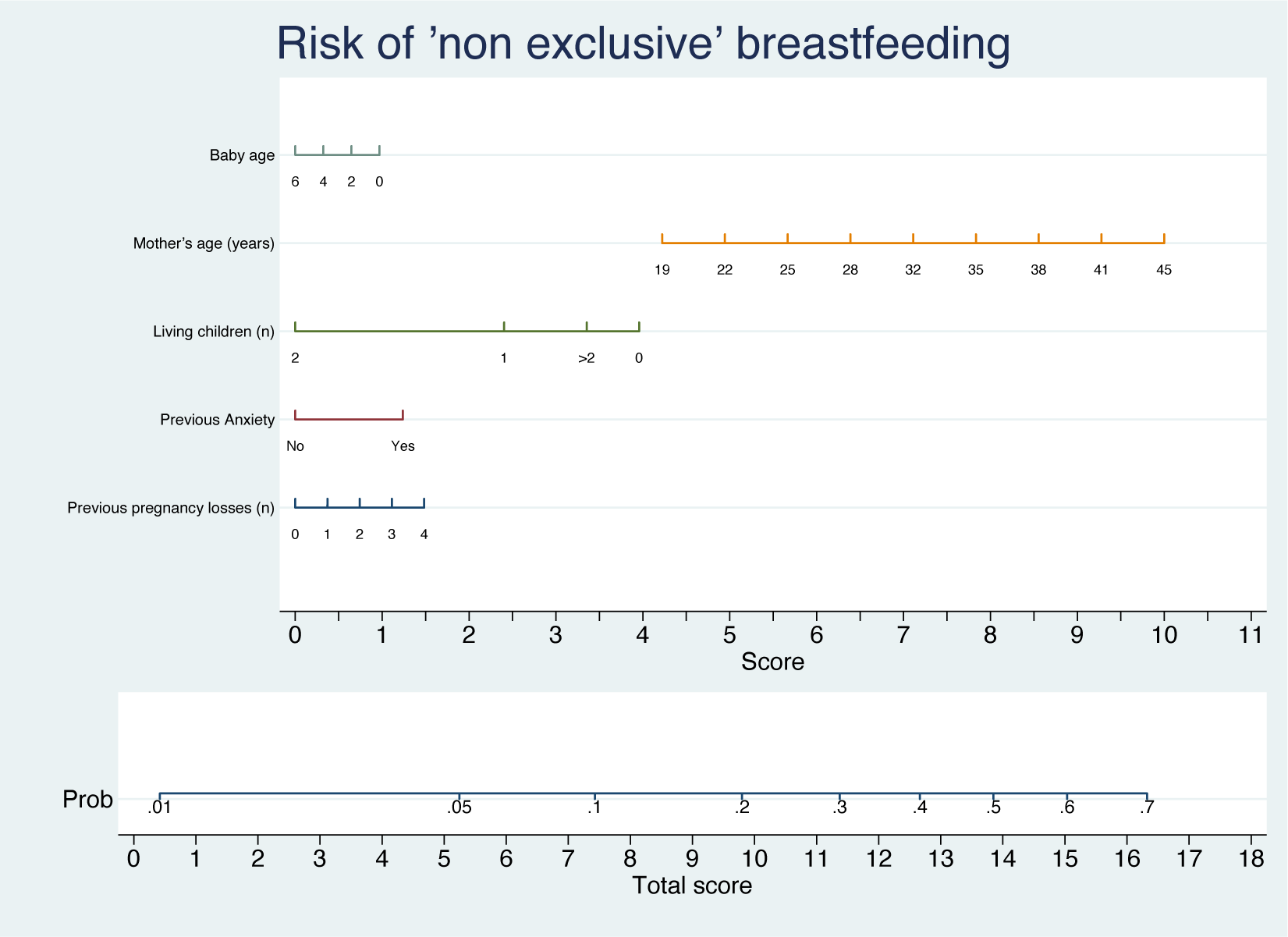

Supplement: Supplementary file 3 [file Image_1.TIF]
